# Global alterations in areas of suitability for maize production from climate change and using a mechanistic species distribution model (CLIMEX)

**DOI:** 10.1038/s41598-017-05804-0

**Published:** 2017-07-19

**Authors:** Nadiezhda Y. Z. Ramirez-Cabral, Lalit Kumar, Farzin Shabani

**Affiliations:** 1 Ecosystem Management. School of Environmental and Rural Science, University of New England, Armidale, NSW 2351 Australia; 2 INIFAP, Campo Experimental Zacatecas, Km, 24.5 Carretera Zacatecas-Fresnillo, 98500 Calera de V.R., Zacatecas Mexico

## Abstract

At the global level, maize is the third most important crop on the basis of harvested area. Given its importance, an assessment of the variation in regional climatic suitability under climate change is critical. CliMond 10′ data were used to model the potential current and future climate distribution of maize at the global level using the CLIMEX distribution model with climate data from two general circulation models, CSIRO-Mk3.0 and MIROC-H, assuming an A2 emissions scenario for 2050 and 2100. The change in area under future climate was analysed at continental level and for major maize-producing countries of the world. Regions between the tropics of Cancer and Capricorn indicate the highest loss of climatic suitability, contrary to poleward regions that exhibit an increase of suitability. South America shows the highest loss of climatic suitability, followed by Africa and Oceania. Asia, Europe and North America exhibit an increase in climatic suitability. This study indicates that globally, large areas that are currently suitable for maize cultivation will suffer from heat and dry stresses that may constrain production. For the first time, a model was applied worldwide, allowing for a better understanding of areas that are suitable and that may remain suitable for maize.

## Introduction

Elevated atmospheric CO_2_ concentrations, global warming and extreme weather events will impact food production, altering the current level of suitability of regions for specific crops. Changes in rainfall patterns and increases in temperature and carbon dioxide levels are likely to have major implications for agricultural productivity, with positive impacts in some regions and negative impacts in others^1–3^. Elevated CO_2_ can improve photosynthetic efficiency, thus increasing the yield of C3 crops and decreasing water consumption through decreases in stomatal conductance in C3 and C4 crops^4^. Conversely, variations in temperature, precipitation and ozone concentrations may affect plant growth and development through increases in abiotic stress^5–8^. Such changes will have important impacts in the quantity and quality of agricultural production, in terms of food security and the welfare of a growing global population^9^.

Maize (*Zea mays* L.) is a major food source for the world and is a high-yield commodity crop, with an average harvested area of 157 million hectares and production of 781 mega tonnes from 2000 to 2014; it is a vital source of food security in many developing countries in Latin America and Sub-Saharan Africa^10, 11^. Furthermore, it serves as forage for the production of biogas^12^. Maize originated in the Mexican Highlands and spread around the world after the colonization of America. Mexico remains one of the main producers, with an average yearly production of 14 mega tonnes from 1961 to 2014, ranked fourth in the world^13–16^. Maize can be produced in an extended range of conditions, from 0 to over 3800 m.a.s.l., and under precipitation levels from 200 mm to 2000 mm^13, 17–19^. Though a variety of abiotic (soil, climate) and biotic (diseases, plagues) stresses affect maize, its main constraints are currently climatic factors and physical characteristics related to soil fertility^12, 20^.

To date, several studies have addressed the possible impacts of climate change on maize, mostly at the regional level and focusing on changes in productivity. Some studies have indicated that temperature increases have a negative effect on maize yield, whereas CO_2_ increases could be beneficial for changes in water availability. However, the level of uncertainty in the CO_2_ results has been consistently high in all research studies^11, 21–23^. This crop is extremely susceptible to drought during the flowering stage, during which the quality of the seed is reduced^6^. Maize drought stress could result in yield losses of nearly 50% in southern Africa^24^. In general, studies have reported a negative impact on maize production that is attributable to increasing temperatures and reduced precipitation^9^. Several institutions around the world have released maize varieties resistant to drought or heat stress to reduce vulnerability^6, 24^.

To assess the potential changes in global maize distribution due to climate change, an appropriate modelling technique should be applied. There are close to one hundred mechanistic and crop niche models that can simulate the potential consequences of climate change on crop production and species distributions. These models differ in their input parameters, protocols and methods^11, 25^. Some widely used distribution models include the following: EcoCrop, which is a mechanistic model that integrates the FAO-EcoCrop database and uses temperature, rainfall and length of the growing season as inputs^26, 27^; MaxEnt, which is an empirical approach that models the potential distributions of species based on presence information of the species of interest^28, 29^; and CLIMEX, which is a hybrid statistical-mechanistic model that is used to estimate the potential abundance and geographic distribution of an organism using climatic data and biological parameters^30^. CLIMEX has been widely used to model the suitability of a variety of organisms, from weeds to insects, at global and regional levels, providing important insights into the ecology of a species^31–34^. An important insight available in CLIMEX is the daily or weekly species’ response to climate variables. Furthermore, the model allows us to explore abiotic constraints, such as heat, cold, dry and moisture stresses^34^.

The objectives of this research were as follows: (i) to employ CLIMEX as a mechanistic species distribution model to assess potential changes in the global distribution of agricultural land for maize cultivation based on shifts in climatic suitability for current versus two time periods, 2050 and 2100 according to projections from two general circulation models (GCMs) CSIRO Mk3.0 and MIROC-H and assuming the A2 emissions scenario; (ii) to identify current maize cultivation regions that are likely to be severely impacted as a consequence of climate change and to identify future stresses; and (iii) to perform a sensitivity analysis to quantify species response to temperature, soil moisture and cold stress changes and to identify the parameters of functional importance to provide a greater understanding of the climatic factors that most impact species distribution.

## Materials and Methods

### CLIMEX description

CLIMEX exemplifies an eco-physiological growth-modelling approach, forecasting shifts in the abundance and distributions of species. The model employs data that are based on climatic information and biological parameters. The locations of climatic suitability are obtained from the species-response functions in CLIMEX, and these functions are based on ecological studies that provide a foundation for the successful modelling of potential species distribution. CLIMEX models the mechanisms affecting species and matches the geographic occurrences with meteorological data. The required monthly climatic data variables include average minimum temperature (T_min_), average maximum temperature (T_max_), average precipitation (P_total_), and relative humidity at 09:00 and 15:00 hours (RH_09:00_ and RH_15:00_) for the specific research locations. The required biological parameters include minimal, maximal and optimal temperatures, as well as soil moisture data for the species. CLIMEX is based on the assumption that a population experiences two seasons. A favourable season produces the temperature and moisture requirements for growth, as described by the annual growth index (GI_A_) that is calculated from the temperature (TI) and moisture indices (MI). An unfavourable season supports no population growth and is represented by four stress indices (cold (CS), dry (DS), hot (HS) and wet (WS)) and their interactions. An annual index of climatic suitability was obtained with the weekly integration of the growth (favourable season) and stress (unfavourable season) indices. This integrated index describes the level of climatic suitability and is known as the ecoclimatic index (EI), which is calculated as follows (E.g. 1):

$$EI=[TI\ast MI]\ast [(1-\frac{CS}{100})\ast (1-\frac{DS}{100})\ast (1-\frac{HS}{100})\ast (1-\frac{WS}{100})]$$

The EI range is from 0 to 100. An EI of 0 describes an unsuitable region for the species to persist and an EI of 100 represents a region with ideal conditions for the species. Such ideal conditions could only exist in a greenhouse or laboratory setting. In CLIMEX, the parameters that describe the response of a species to climate are deduced from its geographic range and phenological observations. Later, the inferred parameters are used to project the potential range in new areas or under new climate scenarios^30, 35, 36^. For a detailed description of the mechanisms of the CLIMEX model, see Sutherst *et al*., (2007) and Kriticos *et al*., (2015). In this study, CLIMEX version 4 was used. The following categories were used for the EI: EI of 0 represents unsuitable regions, an EI from 1 to 10 represents marginal regions, an EI from 10 to 20 indicates regions where large populations can persist (medium suitability) and an EI with values greater than 20 represents a highly favourable region for the species (optimal suitability)^30, 35, 36^.

### Current maize distribution, climatology and climate change scenarios

**Figure 1:**
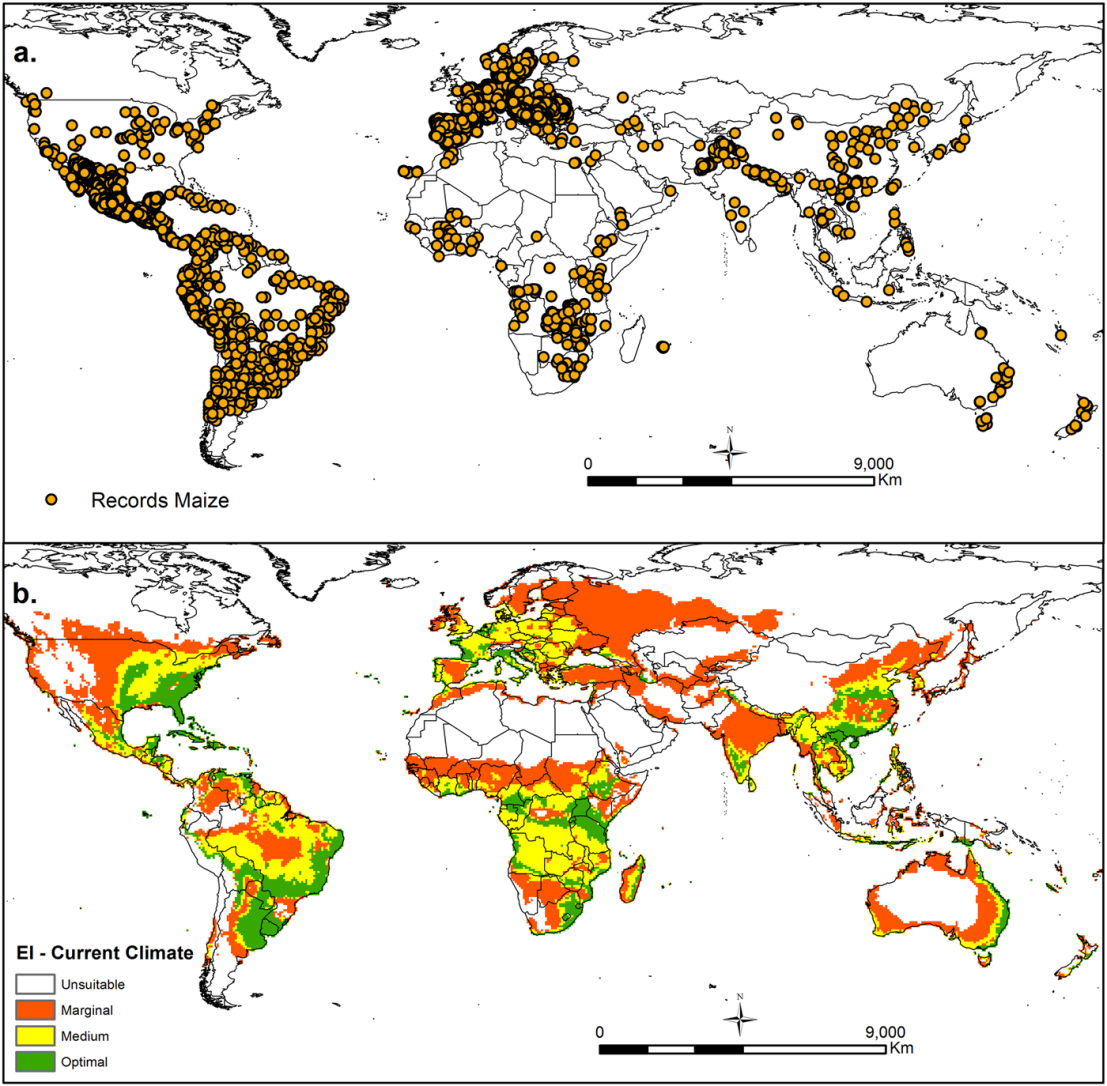
(**a)** The current global distribution of maize (*Zea mays* L.). (**b**) The ecoclimatic index (EI) for the current climate scenario of maize (*Zea mays* L.). ArcMap 10.2 (http://desktop.arcgis.com/en/arcmap).

Current maize distribution data were gathered from the Global Biodiversity Information Facility^37^ and literature resources^11, 17, 38^. The data were verified for biological reasonability; furthermore, duplicate records and those without geographic coordinates were eliminated from the database. Nearly 15,000 records were gathered and used for parameter fitting, with more than 50% of the records representing Mexico. These records geographically represent the current known global distribution of maize (Fig. 1). The use of native and planted (agricultural) distribution records to adjust CLIMEX parameters could generate a better model that more accurately reproduces the potential distribution of the species of study, allowing for the expansion of the fundamental niche^36, 39, 40^. Thus, both native and planted distributions were used to fit the CLIMEX parameters.

CliMond provides 10′ (18.55 km) and 30′ (55 km) global high-resolution data for bioclimatic modelling for CLIMEX and Bioclim. CliMond data are based on WorldClim 10′ (minimum and maximum monthly temperature, monthly total precipitation) and CRU CL2·0 10′ (mean relative humidity). For this study, the climatology of 10′ gridded resolution data was used to model the potential current and future distribution of maize. The data were downloaded in CLIMEX format, containing average monthly maximum and minimum temperatures, average monthly precipitation and relative humidity at 9:00 hours and 15:00 hours^41^. The climate variables were averaged first, after which the species distribution was determined. Historical suitability was modelled with CliMond baseline data, averaging a period from 1961–1990^41^. The future potential distribution was modelled using A2 SRES (Special Report on Emission Scenarios) scenario (business as usual) for 2050 and 2100 based on two GCMs: CSIRO Mk3·0 from Australia^42^ and MIROC-H, developed in Tokyo, Japan^43^. The CLIMEX CliMond climatology data are based on CSIRO and MIROC because these two GCMs have the climate data required for CLIMEX model and have superior performance in areas with a range of climates compared with other GCMs^44^. Data for both models is available on the CliMond website^41^. No other scenarios of the SRES family were included in this investigation, as the A2 SRES scenario incorporates actual CO_2_ emissions levels and population growth trends^45, 46^. The projected global average surface warming by the end of the present century for the A2 scenario is 3.4 °C, with a likely range from 2–5.4 °C^47^, global carbon dioxide emissions are estimated at approximately 30 GtC/yr.^48^ and carbon dioxide concentrations of 846 ppm are expected by 2100^49^. A new report from the IPCC, the AR5 (Fifth Assessment Report) describes four greenhouse gas trajectories, as representative concentration pathways (RCPs) to replace the SRES scenarios^49^. The best equivalent of A2 is the RCP 8.5, representing a higher greenhouse gas emissions scenario^50^. The temperature increase in the period from 2090–2099 is relative to the pre-industrial era for A2 of approximately 6 °C, and the RCP 8.5 is 7 °C^51^. Furthermore, the CO_2_ concentrations by the end of the century for RCP 8.5 are 936 ppm, just above A2. The radiative forcing (W/m^2^) by the end of the century is relatively similar for A2 and RCP 8.5^50^.

### Fitting CLIMEX parameters

Distribution data for India, China and neighbouring countries was not used for parameter fitting, but was reserved for model validation. The reasons for the choices of parameter values are described in detail below. Each parameter was manually and iteratively adjusted until an acceptable visual level of agreement was obtained between the potential and current distribution of maize. The stress indices were first adjusted to maintain the population within the observed distribution limits and to model the core distribution. After adjusting the stresses, the temperature and moisture indices were fixed. All of the CLIMEX parameters were compared with experimental biological data to ensure their validity (Table 1).

**Table 1:** CLIMEX parameter values used for modelling the distribution of maize (*Zea mays* L.).

Index	Parameter	Acronym	Value
Temperature	Lower temperature limit (°C)	DV0	10
	Lower optimal temperature (°C)	DV1	18
	Upper optimal temperature (°C)	DV2	30
	Upper temperature limit (°C)	DV3	35
Moisture	Limiting low soil moisture	SM0	0.1
	Lower optimal soil moisture	SM1	0.7
	Upper optimal soil moisture	SM2	0.9
	Limiting high soil moisture	SM3	1.3
Stresses	Cold stress temperature threshold (°C)	TTCS	7
	Cold stress temperature rate (week^−1^)	THCS	−0.00007
	Heat stress temperature threshold (°C)	TTHS	40
	Heat stress accumulation rate (week^−1^)	THHS	0.01
	Dry stress threshold	SMDS	0.1
	Dry stress rate (week^−1^)	HDS	−0.009
	Wet stress threshold	SMWS	1.3
	Wet stress rate (week^−1^)	HWS	0.001

*Moisture value units are dimensionless indices in proportions of soil moisture holding capacity.

### Stress parameters

#### Cold stress

The cold stress temperature threshold (TTCS) sets the extreme low temperatures, below which the species cannot survive. TTCS is a weekly average of minimum temperatures that are accumulated at a specific rate, known as the cold stress temperature rate (THCS). Maize does not tolerate low temperatures and dies just below the freezing point^5, 13, 52, 53^. Low temperatures affect germination, emergence and vegetative growth. The intensity of the damage will depend on the air temperature and duration of the stress exposure. Maize plants are sensitive to cold temperatures, and frost is detrimental at all stages of plant development, except as dry seed (0 to 6 °C); young plants can be killed at 1 °C^53, 54^ or from the severe cold stress that occurs at 0 °C^5, 55^. A group of cold-induced maize genes (*ZmCOI*) that may affect abiotic stresses was isolated from maize exposed to 6 °C^56^. A review by Garcia and Lopez^7^ found that maize can tolerate temperatures as low as −2 and −3.5 °C, but they did not mention the acceptable duration of low temperatures. Thus, the TTCS was adjusted to 7 °C with a THCS of −0.00007 week^−1^, to allow for the existence of the coldest current locations, such as Norway, Sweden, Finland and northeastern China, but to avoid survival scenarios in Northern Russia.

#### Heat stress

Extreme high temperatures also terminate survival. This factor is modelled through a weekly heat stress temperature threshold (TTHS) and its accumulation rate (THHS). Maize is a thermophilic plant species that can tolerate high temperatures^10, 13, 53^. An extended literature review indicated that extremely high temperatures can cause sterility and reduce yield, with 46 °C being the lethal temperature for maize^5, 57^. Maize can tolerate temperatures below 45 °C, if not, drought stress occurs^13, 54^. Temperatures above 40 °C stop crop development, and no further heat units are accumulated for crop development^57^. Thus, TTHS was adjusted to 40 °C, and the THHS was set to 0.01 week^−1^.

#### Dry stress

This stress starts to accumulate when the conditions are too dry for the species. The weekly dry stress threshold (SMDS) accumulates at a given rate (HDS). Maize is susceptible to drought, especially during flowering, tasselling, silking and pollination, as well as during the grain-filling stage during which a lack of water can cause losses of nearly 90%, with little or no grain yield^6, 12, 13, 57^. Water shortages can restrict cell division and growth in maize^53^. Doorenbos and Kassam (1979) mentioned that with sufficient water availability, maize can tolerate dry atmospheric conditions and, during the vegetative and ripening periods, may be relatively tolerant of water deficits^13, 54^. Since maize can tolerate water shortages, the SMDS was set at 0.1 (the permanent wilting point), at a stress accumulation rate (HDS) of −0.009 week^−1^.

#### Wet stress

Extreme wet conditions can produce wet stress. This stress accumulates weekly once the soil moisture has passed a wet stress threshold (SMWS) at a given rate (HWS). Waterlogging may reduce growth, photosynthesis and cause a loss of biomass production or even death. Older maize plants are more tolerant to waterlogging than younger ones^6, 58^. The phenological stages that are more susceptible to waterlogging precede those of tasselling and flowering. Damage to the roots, due to the accumulation of toxic bioproducts, affect yields^6, 13, 54^. Maize prefers aerated and well-drained soils^13, 54^, although some varieties can produce adventitious roots and tolerate waterlogging^20^. Therefore, a SMWS of 1.3 was used with an accumulation rate (HWS) of 0.001 week^−1^.

### Growth-related parameters

#### Temperature index

This weekly index describes the response of the crop to the daily temperature cycle, ranging between a lower temperature threshold (DV0) and an upper temperature threshold (DV3); optimal temperatures occur between the lower optimum temperature (DV1) and the upper optimum temperature (DV2). The most common base temperature for maize growth is 10 °C^5, 7, 10, 53, 59–61^. Thus, the DV0 was set at 10 °C. Maize requires high optimal temperatures for germination and growth^53^. The influence of temperature on the germination and elongation of maize radicles has been tested^62^. The elongation rate peaked at 30 °C and stopped at the extremities of 9 °C and 40 °C^62^. At 20 °C, early and medium grain varieties take between 80 and 140 days to mature. Optimal temperatures for germination are 18 to 20 °C^13, 54^. The optimal range during daylight is from 25 to 33 °C, and at night it is 17 to 23 °C. The optimal temperatures for the whole crop season are between 20 and 22 °C^63^. The DV1 was set at 18 °C and DV2 was set at 30 °C as a compromise between the varied values in the literature^5, 57, 59–61, 64^. Research has established temperatures ranging from 36 to 40.2 °C as maximal for growth development in maize^5^. Temperatures higher than 35 °C reduce dry matter accumulation during grain filling^11, 63, 65^. Thus DV3 was set at 35 °C.

#### Moisture index

The hydrological model integrated in CLIMEX represents the effects of rainfall and evaporation within the species, termed the ‘moisture index’^36^. Maize is most susceptible to water stress at flowering^66^. A study to determine the sensitivity of maize to water stress found that the threshold of the soil moisture stress index was between 0.20 and 0.30^67^. The limiting low soil moisture (SM0) was set to 0.1 to represent the permanent wilting point^68^, a value that is also consistent with the dry stress threshold (SMDS). The lower (SM1) and upper (SM2) soil moisture values were adjusted to 0.7 and 0.9, respectively. The limiting high soil moisture (SM3) was set at 1.3 to maintain consistency with the wet stress threshold (SMWS). Excessive moisture is a main constraint in regions such as Southeast Asia and India^20^. These values provided the best fit for current maize distribution. The CLIMEX parameters are summarized in Table 1.

Once the best fit was obtained, the final model was run for historical and future scenarios and maps were generated. Maps for the four stress indices were also obtained for current and future scenarios.

### Parameter sensitivity of CLIMEX values

The sensitivity analysis determines how the accuracy of the parameters affect the model output when applied on an individual basis. A parameter sensitivity analysis runs the model repeatedly, altering individual parameters successively to exceed their fitted values. This analysis was carried out with the global CliMond database. Variables with a greater effect on the model output are described as ‘sensitive’, whereas those that have no impact on the model output are termed ‘insensitive’^34^.

### Methods of validation

Occurrence data from India, China and neighbouring countries was not used in the fitting process, but was reserved for the validation process. Once the parameter fitting was satisfactory, the validation area was verified to check the performance of the model in this region. Furthermore, a cross validation that is incorporated in CLIMEX was performed, using two maps of current maize distribution based on productivity^69, 70^. The agreement between the modelled historical potential distribution and the seasonal phenology of the modelled species in different areas provided the cross validation for the model^34^.

### Estimating land areas for various suitability classes

The Food and Agriculture Organization (FAO) has an agricultural statistical database that is available for download. Information on the average global maize yields from 1983 to 2013 was obtained from this database^71^, which was also used to identify the major maize-producing countries.

Category levels of suitability (marginal, medium, and optimal) and unsuitable areas, as reflected by the EI values, were determined according to continent, and for each of the five major maize producing countries. The raster images were re-projected using Behrmann projection to obtain the estimated sizes of the regions for the spatial analysis^41^.

## Results

### Validation methods, parameter sensitivity

The modelled distribution (Fig. 1b) shows an acceptable match with the current known distribution of maize (Fig. 1a). Approximately 96% of the occurrence records are classified in the marginal to optimal categories, while 88% of the occurrence records for the validation area (India, China and neighbouring countries) are located within the modelled marginal to optimal areas. The global distribution of You *et al*., (2014) and Leff *et al*., (2004), used for cross validation, indicates broad agreement with our maize model under historical conditions. These validations confirm the optimal performance of the present model and the validity of the parameter values selected.

The parameter sensitivity indicates that a limiting low moisture (SM0) of 5.3% and cold stress temperature rate (TCHS) of 3.5% impact are the most sensitive parameters in the modelled potential range (Table S1). It is important to note that the range change is at the global level.

### Current scenario

The modelled results indicate medium to optimal climatic suitability for the eastern United States, Mexico, Brazil, Argentina, some other South American countries, Central and Southern Africa, most of Europe, Southern India, Eastern China and the Australian coasts. The Sahara Desert, central Australia, most of Canada, Mongolia and a large part of Russia are among the countries or territories with unsuitable conditions. The current modelled projection results show that over 60% of the African continent has some degree of climatic suitability for maize, compared with 77% of South America. Europe was projected to have the largest area of climatic suitability for maize cultivation, with almost 82% coverage (Table 2).

**Table 2:** Projected areas of unsuitability and suitability for maize by continent under current climate conditions and percentage of areas. Percentage values indicate changes in the suitability of areas under future projected climate from the current climate.

	Current climate scenario				Percentage change in areas under a future projected climate scenario			
	Total area (10^6^ km^2^)		Percentage		CSIRO		MIROC	
					2050	2100	2050	2100
	EI = 0	EI > 1	EI = 0	EI > 1	EI > 1	EI > 1	EI > 1	EI > 1
Africa	11.28	18.55	37.8	62.2	−11	−36	−7	−29
Asia	28.81	15.88	64.5	35.5	4	7	4	11
Europe	1.82	8.10	18.3	81.7	11	16	10	14
North America	13.91	10.24	57.6	42.4	10	19	10	20
South America	4.07	13.69	22.9	77.1	−5	−43	−5	−43
Oceania*	4.25	3.82	52.7	47.3	−2	−30	−5	−10

*Oceania = Australia and New Zealand.

EI = 0 is unsuitable area. EI > 1 includes three suitable categories (marginal, medium and optimal).

Note that these are the total suitable projected areas and not the actual areas under maize cultivation.

### Future climate projections

**Figure 2:**
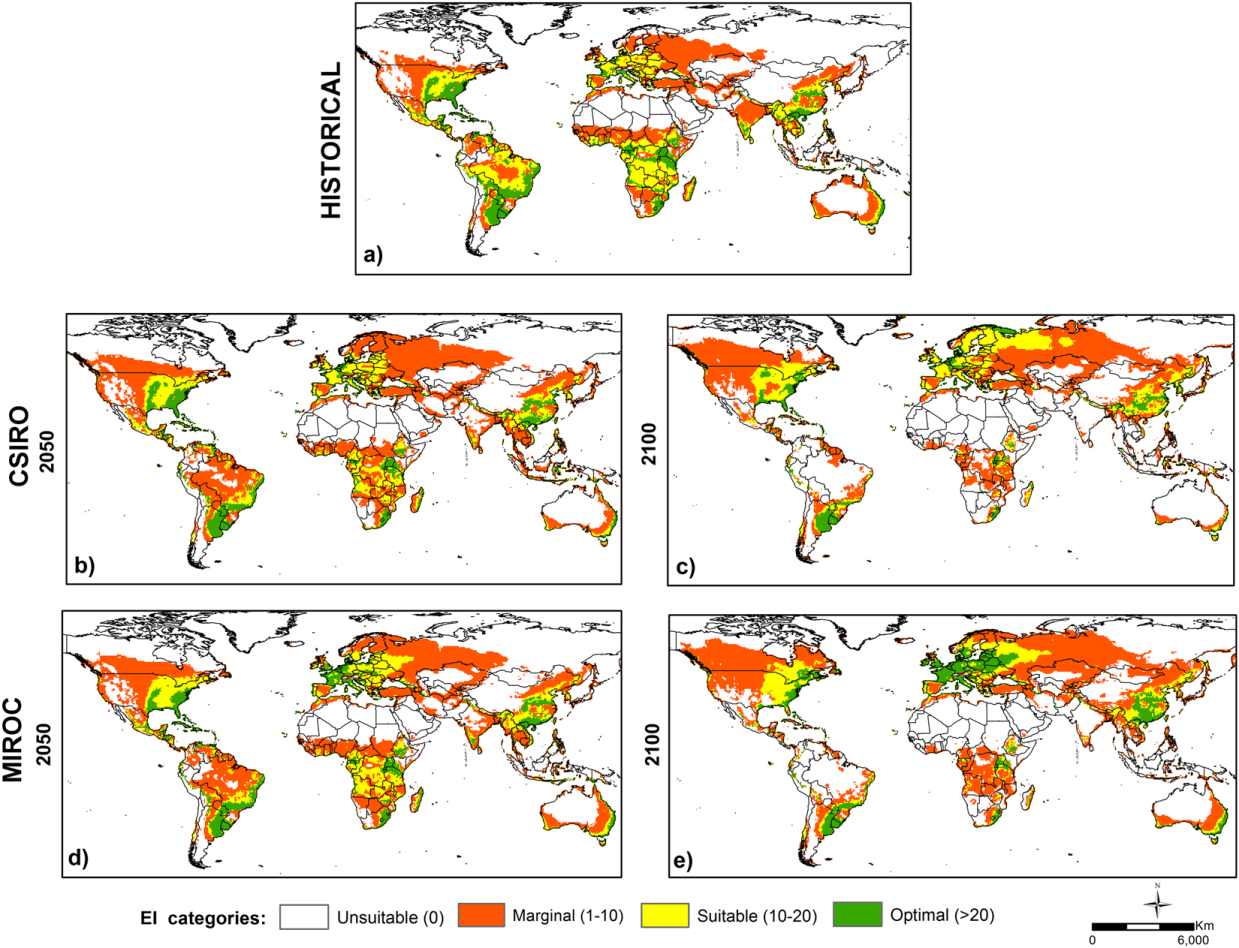
The Ecoclimatic Index (EI) of future climate conditions for maize (*Zea mays* L.) under the SRES A2 (resembling RCP 8.5) in CSIRO: (**a**) Historical (**b**) in 2050 (**c**) in 2100; and in MIROC (**d**) in 2050 (**e**) in 2100. ArcMap 10.2 (http://desktop.arcgis.com/en/arcmap).

The future scenarios project a loss of climatic suitability area for maize in Sub-Saharan Africa and Latin America, but an expansion in the northern hemisphere, particularly in Europe (Fig. 2). The following section describes the results for 2050. Both GCMs project similar trends for America, Africa, Asia and Oceania. For South America, a shift in climatic suitability can be observed, with previously medium areas changing to marginal suitability. In North America, an increase in areas of marginal suitability have also been projected (Fig. 2a,c). Both GCMs project a loss of approximately 5% of general suitability in South America and an increase of approximately 10% in general suitability for North America (Table 2). In Africa, areas of climatic suitability for maize cultivation are projected to contract, with shifts from medium to marginal climate suitability in Angola, Zambia, Mozambique and Congo (Fig. 2a,c). The CSIRO model indicates a reduction of 11% in areas with climatic suitability, with this figure at 7% in the MIROC model (Table 2). In Asia, including the Middle East, climatic suitability for maize is projected to remain similar to current conditions. In India, marginal areas will become unsuitable, whereas in South East Asia, most medium suitability areas will change to marginal suitability by 2050. Bangladesh, Burma, Thailand, Laos and Cambodia will see a decrease in medium suitability areas and an increase in marginal suitability areas. An increase of 4% in climatically suitable areas for maize cultivation is projected under both GCMs for Asia, especially China (Fig. 2a,c) (Table 2) (Fig. S1).

In Australia, areas of marginal suitability are projected to decrease in New South Wales, Queensland and Victoria under CSIRO (Fig. 2a). Larger decreases in areas with a medium climate for maize are projected under MIROC (5%) (Table 2). For Europe, the modelled projections under the two GCMs indicated slight differences. MIROC projected larger increases in the medium category compared to CSIRO, with medium areas extending to Russia and an increase in optimal suitability for England, France, Germany, Denmark, Netherlands, Poland, Slovakia and the Czech Republic. Both GCMs projected a change from unsuitable to marginal suitability in the Nordic Countries (Fig. 2a,c). The projected percentage of change under CSIRO and MIROC is similar, with increases of 10%. However, CSIRO projected an increase in the marginal suitability category, whereas under MIROC the increase corresponds mostly to optimal areas (Table 2).

Interestingly, Africa and Asia exhibited similar trends under both GCMs for 2100. For Africa, there is a considerable reduction in climatic suitability for maize. The Democratic Republic of Congo, Angola, Zambia, Mozambique Central African Republic, Cote d’Ivoire, Ghana, Togo and Nigeria may have small areas with marginal suitability remaining by the end of this century. Ethiopia, Kenya, Uganda, Tanzania and Madagascar could be some of the few remaining African countries with medium and optimal suitability by 2100 (Fig. 2b,d). Unsuitable areas will increase under CSIRO, with a 36% increment, compared to the 29% indicated under MIROC (Table 2). In Asia, reductions in climatic suitability for maize cultivation are projected for India, Malaysia, Singapore, Philippines and Indonesia. Mongolia and Southern Russia may become marginally suitable by 2100. Eastern China is projected to increase in optimal suitability, particularly under the MIROC scenario (Fig. 2b,d). The increase in suitability is low for Asia, at 7% under CSIRO and 11% under MIROC (Table 2).

By 2100, America, Europe and Oceania indicate some differences may occur between the two GCMs. Canada and the USA are projected to become more suitable for maize, with MIROC exhibiting larger increases in the south-eastern parts of Canada compared to CSIRO. Most Latin American countries are projected to experience a reduction in maize suitability, mainly in the optimal areas. Mexico, Brazil, Argentina, Paraguay and Peru may preserve some optimal and medium suitability areas. Interestingly, Uruguay may remain as optimally suitable until 2100 (Fig. 2b,d). Climatic suitability in North America is projected to increase by 19% and 20% under CSIRO and MIROC, respectively. South America is projected to decrease in suitability by 43% under both GCMs (Table 2). All of the European countries are projected to be suitable for maize to some extent. The MIROC model projects an important increase in optimal suitability in Scotland, England, France, Germany, Poland, Italy, Belarus and Russia. An increase in marginal suitability is expected for the Nordic countries and western Russia. CSIRO indicates a shift from marginal to medium suitability in northwestern Russia and in the Nordic countries, and an increase in the medium category in England and Italy (Fig. 2b,d). The percentage change in these areas is similar under both GCMs, with 16% under CSIRO and 14% under MIROC (Table 2). Finally, in Oceania, CSIRO predicts a three-times greater increase in unsuitable areas in Australia (30%) compared with MIROC (10%). New Zealand remains similar to current conditions (Fig. 2b,d) (Table 2).

**Figure 3:**
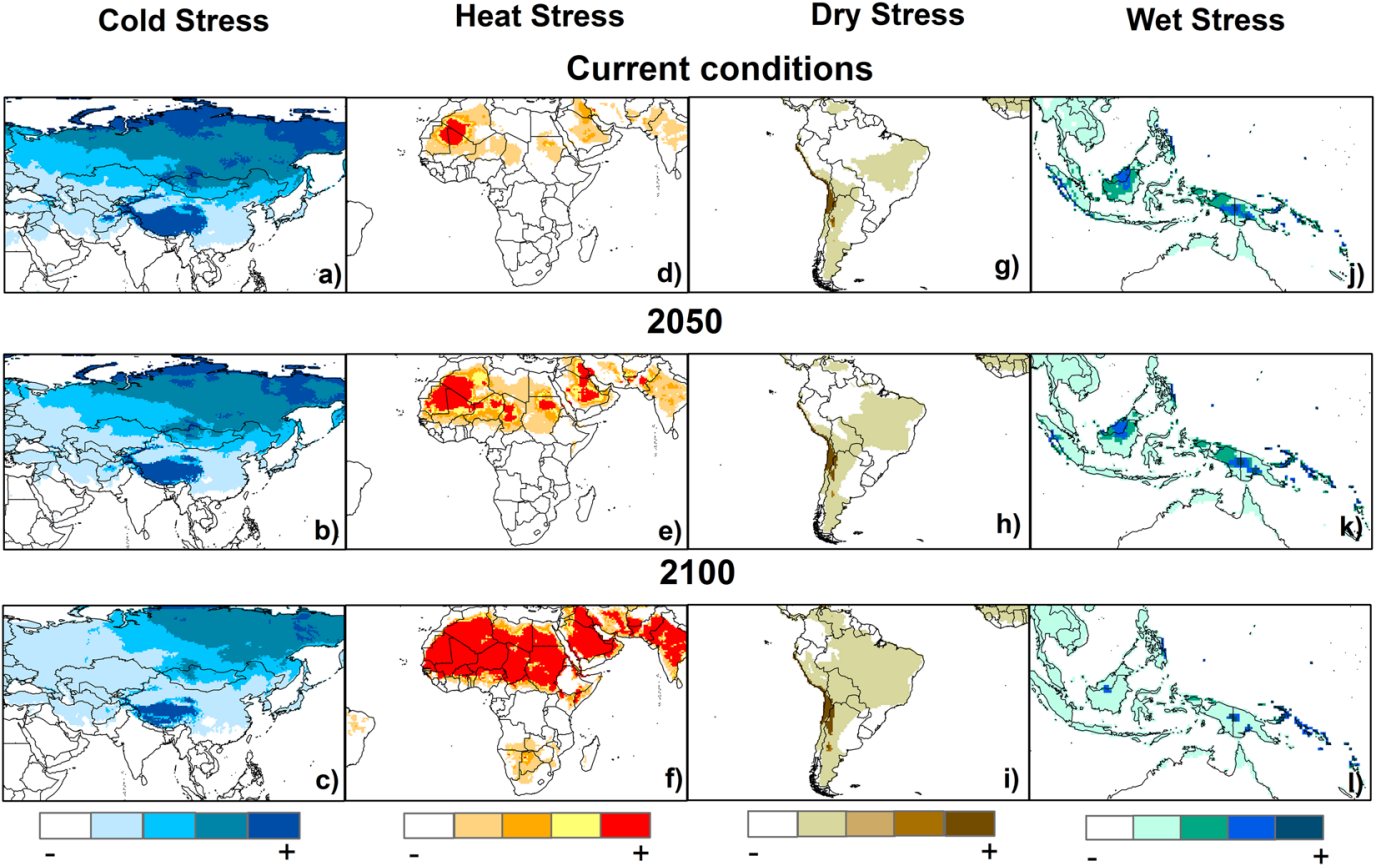
Projections of cold stress in Europe and Asia; (**a**) current scenario, (**b**) 2050, and (**c**) 2100; heat stress in Africa: (**d**) current scenario, (**e**) 2050, and (**f**) 2100; dry stress in South America: (**g**) current scenario, (**h**) 2050, and (**i**) 2100; and wet stress in Southeast Asia: (**j**) current scenario, (**k**) 2050, and (**l**) 2100. ArcMap 10.2 (http://desktop.arcgis.com/en/arcmap).

The stresses were mapped to visualise the changes from the current scenario to the future projections. Cold stress is projected to decrease poleward at a global scale. Figure 3 shows the reduction in Europe and Asia. Heat stress may increase as a result of climate change, mainly in South America, Africa, parts of Asia and Australia. An illustration of the increase in heat stress is shown for Africa and some Asian countries in Fig. 3. Regarding dry stress, a worldwide increase is projected in accordance with a decrease in wet stress (Fig. 3). See also Fig. S3.

### Current and future climate situation in the major maize-producing countries

According to the FAO, during the period from 1983 to 2013^71^, the USA, China, Brazil, Mexico and Argentina were listed as the major maize producers. These countries are responsible for approximately 70% of the global maize production, with the USA and China together producing more than half of the total.

**Figure 4:**
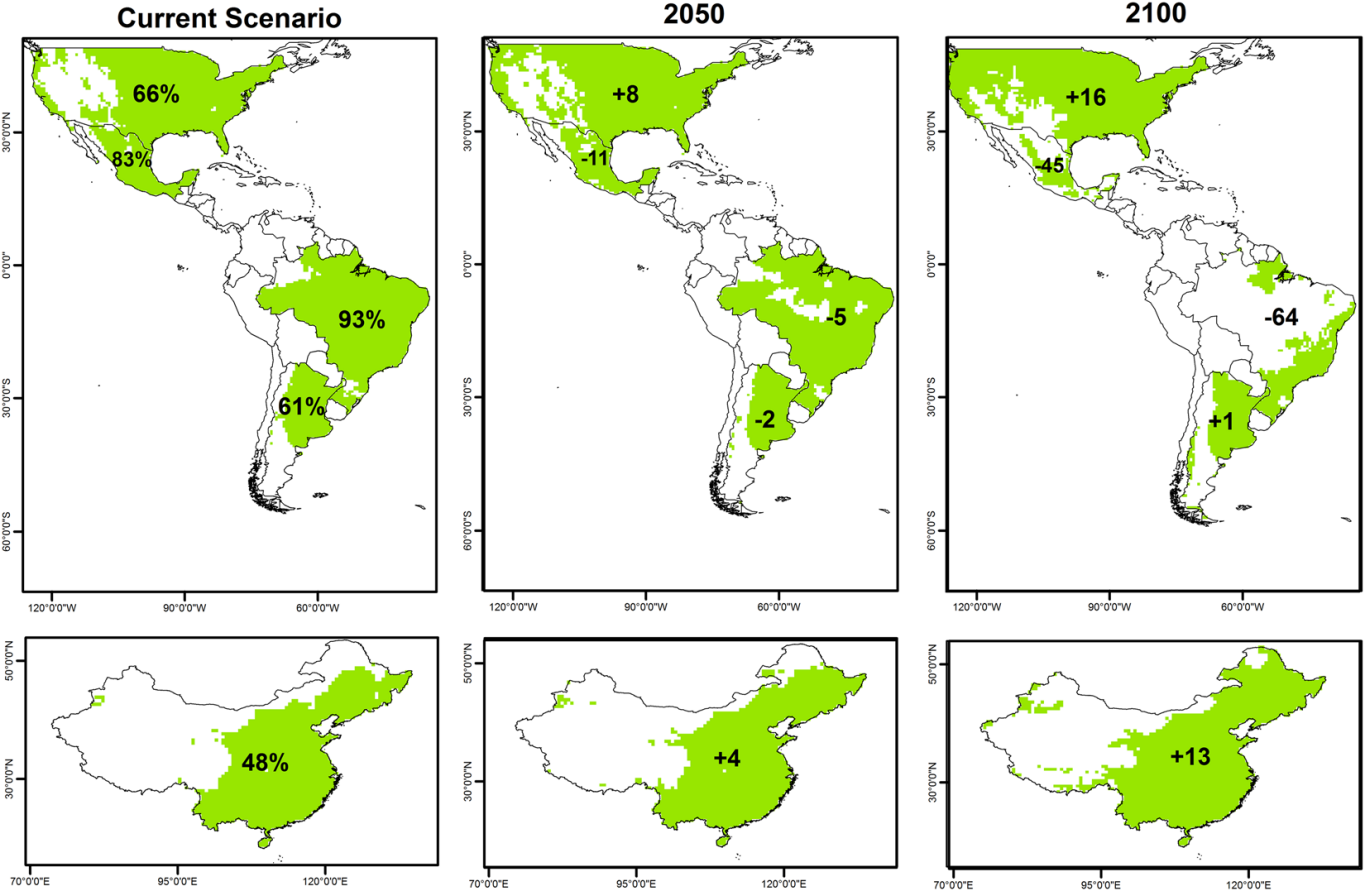
Changes in areas of climatic suitability for maize among the current five major maize producers. For example, under current conditions China is modelled to have 48% of its territory with some climatic suitability for maize cultivation, with an increase of 4% expected by 2050 and 5% by 2100. ArcMap 10.2 (http://desktop.arcgis.com/en/arcmap).

Under current climate conditions, approximately 50% of the global area, with some degree of suitability for maize, falls into the territories of these five major producers. Argentina has a large area of optimal suitability, whereas Brazil has larger areas in the medium category. The three remaining countries have mainly marginal suitable areas for maize (Table 3). Under projected future climate conditions, a reduction in optimal areas in Argentina is indicated. In the 2050 scenario, Argentina will see a reduction in climate suitability for maize, but may recover some by 2100 (Table 3). It is projected that cold and dry stresses may decrease in Argentina by 2100, and heat stress is not projected to occur in Argentina. Brazil, between 2050 and 2100, may see a radical reduction in climate suitability, principally in the medium and optimal categories due to an increase of dry and heat stresses, even when wet stress is expected to decrease and no changes are expected in cold stress. South of the Rio Grande do Sul, Parana and Sao Paulo remain as areas of optimal or medium suitability. A decrease of 64% in suitable areas is projected for Brazil (Table 3). China remains similar to current conditions in the 2050 scenario under both GCMs, with an approximately 5% increase in suitable areas. Under CSIRO for 2100, suitability in eastern China could increase by 13%, whereas under MIROC, this figure is as high as 20% (Table 3). For China, cold and wet stresses may decrease in future scenarios, whereas dry stress is projected to increase slightly. For Mexico, an increase of greater than 60% in unsuitable areas is projected by 2100 under both GCMs. Areas with medium suitability may be considerably reduced under future climate scenarios (Table 3). The USA exhibits a reduction in optimal areas, with an increase in areas in the medium category. Under CSIRO, a 16% increase in suitable climatic areas is projected for the USA, with MIROC indicating a more conservative increase of 9% (Table 3) (Fig. S2) In Mexico and the USA, cold and wet stresses are projected to decrease, contrary to heat and dry stresses, which will increase. Dry stress will cover almost the entire territory of Mexico by 2100, but only the western part of the USA. Figure 4 indicates the percentage of suitable areas for maize cultivation under the current scenario for the major five maize producers. The future scenarios indicate increases or decreases in this area for the major groups in relation to the current scenario.

**Table 3:** Projected unsuitable and suitable areas for the five major producers of maize under current climate conditions and coverage. Percentage changes indicate alterations under the future projected climate in relation to the current climate.

	Current climate				Percentage changes in areas under future projected climate change			
	Total area (10^6^ km^2^)		Percentage		CSIRO		MIROC	
					2050	2100	2050	2100
	EI = 0	EI > 1	EI = 0	EI > 1	EI > 1	EI > 1	EI > 1	EI > 1
Argentina	11.05	16.92	39.5	60.5	−2	1	−2	−1
Brazil	6.41	78.81	7.5	92.5	−5	−64	−7	−63
China	48.78	45.23	51.9	48.1	4	13	5	20
Mexico	3.31	16.15	17.0	83.0	−11	−45	−12	−47
USA	32.33	62.75	34.0	66.0	8	16	6	9

EI = 0, unsuitable area. EI > 1 includes three categories: marginal, suitable and optimal suitability.

Note that these are the total suitable projected areas and not the actual areas under maize cultivation.

Changes in areas of climatic suitability for maize among the current five major maize producers. For example, under current conditions China is modelled to have 48% of its territory with some climatic suitability for maize cultivation, with an increase of 4% expected by 2050 and 5% by 2100. ArcMap 10.2 (http://desktop.arcgis.com/en/arcmap).

## Discussion

### Validation and parameter sensitivity

This study was based on the biological and climatic requirements that cover all of the maize mega-environments. Taking into account these requirements, the model provides a good fit with the current global distribution of maize, including the validation area, which provided data that was not used to determine the bioclimatic requirements (India, China and neighbouring countries) (Fig. 1). In this study, the model represented approximately 96% of the occurrence records worldwide during the calibration phase and 88% of the occurrence records within the validation area matching the suitable areas for maize cultivation estimated using CLIMEX modelling.

The cross-validation performed with maps from MapSPAM (Spatial Production Allocation Model)^70^ and research into the geographic distribution of major crops^69^ generally matched with the modelled current global distribution of this study. The areas with high maize production from the MapSPAM map for rainfed conditions agree with the areas of optimal suitability in our model, such as the Maize belt of the USA^70^. This cross-validation provides further verification of our model, indicating consistency between the potential geographical distribution of the CLIMEX model and the global seasonal phenology of maize provided by You *et al*., (2014) and Leff *et al*. (2004).

The low percentage in the range change in the sensitivity analysis provides confidence about the chosen parameters^34, 72^. In summary, all of these tools support a satisfactory level of confidence in the maize model performance for current and future climate scenarios.

### Current scenario

Even where the areas of climatic suitability estimated for the current conditions in this research match the known distribution, the CLIMEX maize model differs from other projections. The EcoCrop mechanistic model projects higher maize suitability in Africa, Australia and India and lower suitability in the Maize Belt of the USA, Europe and China under current conditions^27, 73^. This may be because the EcoCrop temperature thresholds differ from those used in this research. For example, the EcoCrop maximum temperature was set at 47 °C, whereas in this study, a value of 35 °C was used. This difference may be a result of the high level of biological diversity and climatic requirements of maize^18^ and of the model choice; for example, the Maize Belt of the USA has an average temperature between 26 to 34 °C, whereas in Ethiopia, Mexico and the Andean zone, the range is between 18 to 24 °C^18^. Furthermore, the EcoCrop niche projection for the USA does not predict high suitability in the Maize Belt of the USA; however, this region produces more than 30% of the world’s maize yield, mainly under rainfed conditions^18^. In contrast, the present model projects from medium to high suitability in this region.

### Future scenarios

Regions with low temperatures will become warmer and more suitable for crop cultivation under predicted climate changes in the future as a consequence of new areas becoming available for agricultural production^27, 47^. These findings agree with the results of the present study, which indicate the expansion of climatic suitability for maize in cooler regions (northern latitudes) such as North America and Northern Europe, in agreement with a reduction in cold stress in future scenarios, under both GCMs for these regions. Currently, maize does not have optimal conditions in Northern Europe, but despite climate constraints, it has become established as a competitive crop in countries such as Denmark^74^. The Nordic countries started to cultivate maize mainly for silage, due to the short growing season and the low temperature limits for grain production. These countries are projected to increase maize cultivation into more northern latitudes as a result of the use of new hybrids and/or the climate change impacts^75, 76^. This change in suitability may increase the competitiveness of maize in cooler regions and may create conditions for grain production, not only silage.

The CERES (Crop Environment Resource Synthesis) crop model predicts a reduction in production of almost 12 million tons per year; representing 10% of the current production of Latin American and African countries by 2055, using data based on four maize varieties in rainfed conditions^10^. Such a prediction bears a direct relationship to the results of the present study in terms of the projected reduction of suitability in Latin America and Africa. Though production yields were not estimated in the present study, the level of climatic suitability is directly related to changes in maize production. Another study, using the SRES and HadCM3 GCM, with production functions that incorporate temperature, precipitation and the response to CO_2_, projects a large reduction (30%) in climatically suitable areas for maize in Africa^1^, bearing a similar relationship to the reduction of 32.5% projected in this study on this continent.

The A2 scenario (resembling RCP 8.5) describes increased rainfall and moderately increased temperature in most developed countries^2, 47^. Conversely, developing countries, mainly in the Southern Hemisphere, face decreased rainfall and greatly increased temperatures^1^. Such is the case for Honduras and El Salvador, which may suffer maize yield losses greater than 10% by 2020 due to projected global-warming factors^77^. It is crucial in that the availability of precipitation is rated as the most important constraint in maize productivity^12^. At the global level, the results of this research indicate a significant reduction in climatic suitability for maize cultivation in developing countries and expanded or greater suitability for some developed countries (USA, Canada, Europe and Australia). These previous results indicate comparative agreement with increases in heat stress over Africa and dry stress in Latin America, as noted in the projections in this study. Moderately higher temperatures, shortening of the growing cycle resulting in reduced dry matter in grain maize, and the increase in heat stress and dry stress are likely to be responsible for reduced yields and degraded climate suitability^11, 12, 63^.

As mentioned earlier, the top maize producers are the USA, China, Brazil, Argentina and Mexico^71^. The present model projects an increase of up to 16% in climatic suitability for maize in the USA, and China is also projected to have increased climate suitability. In China, maize is one of the major crops and is grown over an extended range of climatic conditions, from cold temperate to sub-tropical. During recent decades, there has been a tendency to increase the sown area and consequently the production yield in China^38, 78^. The more productive regions in China and the USA are those projected to increase in suitability, as shown by the present results. In contrast, our results indicate a reduction in areas of climatic suitability for maize in Brazil and Mexico. Some researchers have suggested that the availability of agricultural areas will be reduced due to climate change in northeastern Brazil and, as a consequence, productivity could be reduced^79^. By 2050, a 30% reduction in maize production is predicted for Brazil^80^; the present study projects a reduction of climate suitability of 5% by 2050 and a dramatic increase by the end of the century to 64%. Similarly, reports from Mexico suggest a reduction in rain-fed maize production and areas suitable for its cultivation^81, 82^. Finally, the present modelling predicted no significant change in Argentina for maize suitability; whereas some authors have predicted an increase in production due to climate change effects in Argentina^83, 84^. Travasso *et al*., (2009) modelled maize production in Argentina with the regional model MM5/CIMA and predicted increases in yield with the inclusion of CO_2_ effects, whereas without the inclusion of CO_2_ effects the production would reduce by 9% under SRES A2.

This future global scenario has important implications for food security in developing countries. However, development of new technologies and management adaptation could mitigate the impact of climate change on maize cultivation. For example, the development of stress tolerant varieties can reduce losses due to heat, drought, frost and hail in areas where conditions become unfavourable^11, 78^, and through adaptation measures, such as changes in planting date and water saving techniques^84^. Currently, varieties with elevated levels of drought tolerance have been introduced successfully in Asia and could be used in regions with similar characteristics^6^. Without investment and research into adaptation, developing countries are likely to suffer the negative impacts of climate change, with massive implications for food security among the populations of these countries^85^. It is important to mention that regardless of the differences between GCMs, the model outputs follow a similar trend of future climate predictions. This is because the fundamental basis is the same, despite some differences^47^.

### Constraints of the study

Recently, in the AR5 report, the IPCC adopted representative concentration pathways (RCP) to replace the SRES. Moreover, new general circulation models (GCMs), such as the Coupled Global Climate Model (CGCM3), MIROC3.2, CNRM-CM3, CSIRO Mark 3.0, CM2.0-AOGCM, FGOALS1.0_g, INMCM3.0, Parallel Climate Model (PCM) and HadCM3 are becoming available. The new data from the RCPs can be used in various correlative species distribution models, such as MaxEnt, the Generalized Linear Model, Random Forest and Boosted Regression Trees; however, the available data for CLIMEX are only from two SRES (A1B and A2) and two GCMs (CSIRO Mk3.0 and MIROC-H). Shabani *et al*.^86^ explains the differences between performance of different correlative and mechanistic species distribution models.

We would also like to note that the CLIMEX model does not incorporate certain limiting abiotic and biotic factors, such as soil type, pests, pest interactions and weed impacts. Thus, one criticism of this study is that estimates of technical feasibility fail to identify the areas where it would be economically desirable to cultivate the species. Inclusion of non-climatic factors, such as topography, soil taxonomy, physicochemical properties of soil, and land usage on the national level, are required and could be included in future studies of maize suitability projections, either at global or regional levels. Furthermore, in the present study, the potential genetic progress of the species was not taken into account.

## Conclusion

This study assessed the potential impacts of increased greenhouse gas emissions on climate suitability for maize cultivation, and consequently its distribution around the world. The use of CLIMEX allowed for a deeper understanding of the stress factors reducing the climate suitability of a species under future scenarios. Moreover, the high percentage of records in the validation area, as well as the sensitivity analysis, reduced the uncertainty in the CLIMEX model. Our main findings indicate that areas of climatic suitability for maize production are projected to increase in many developed countries (poleward areas), such as the USA, which is a major production area, due to a reduction in cold stress, whereas reductions in suitability are projected mainly for developing countries, such as Mexico and Brazil, which are two important production areas, and in many African countries, due to an increase in heat and dry stresses. Millions of people in Africa and Latin America depend on maize as their staple crop. Smallholders in these countries, as well as a portion of the population they feed, may be affected by the reduction in maize suitability, jeopardizing food security. The USA and China, two of the main maize producers (60%), are likely to see an increase in areas suitable for maize cultivation. This study indicate that heat and dry stresses may limit maize suitability in the future for countries between the tropics of Capricorn and Cancer; however, agricultural research and the genetics of plant cultivars could mitigate some of the negative effects of climate change for maize with new varieties that are tolerant to heat and drought conditions.

## Electronic supplementary material

Supplementary Material
