# Supplementary material for: Global alterations in areas of suitability for maize production from climate change and using a mechanistic species distribution model (CLIMEX)
Source: Sci Rep. 2017 Jul 19;7:5910. doi: 10.1038/s41598-017-05804-0 (PMC5517596; doi:10.1038/s41598-017-05804-0)
Supplement: Supplementary file 1 — Supplementary Material [file 41598_2017_5804_MOESM1_ESM.doc]

**Global alterations to areas of suitability for maize production, based on climate change and a mechanistic species distribution model (CLIMEX).**

Nadiezhda Y. Z. Ramirez-Cabral*^1,2^. Lalit Kumar^1^. Farzin Shabani^1^

^1^Ecosystem Management. School of Environmental and Rural Science, University of New England. Armidale, NSW 2351. Australia. ^2^INIFAP. Campo Experimental Zacatecas. Km. 24.5 Carretera Zacatecas-Fresnillo, 98500 Calera de V.R., Zacatecas, Mexico.

Supplementary Information


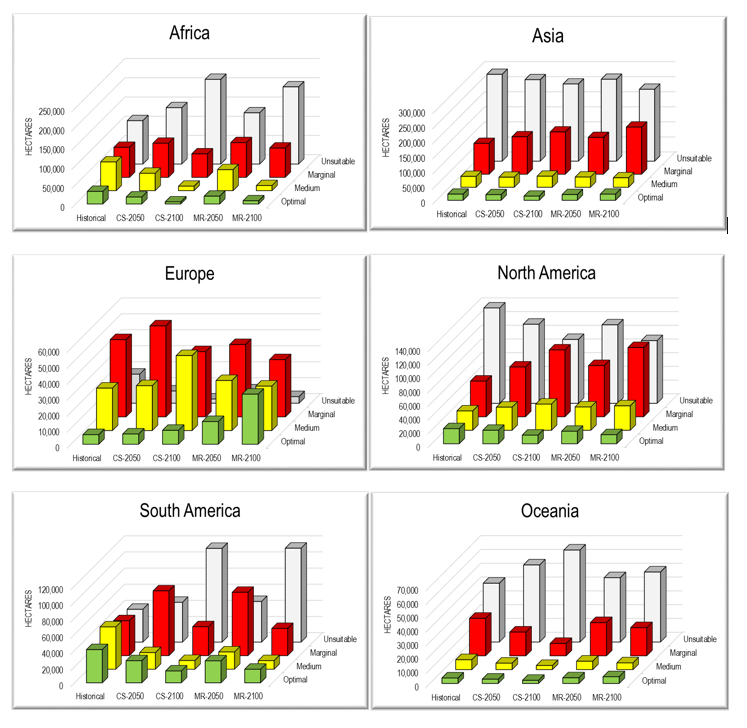
Figure S1. Projected areas of unsuitability and suitability for maize by continent under current and future climate conditions based on CS and MR models and the A2 scenario through CLIMEX


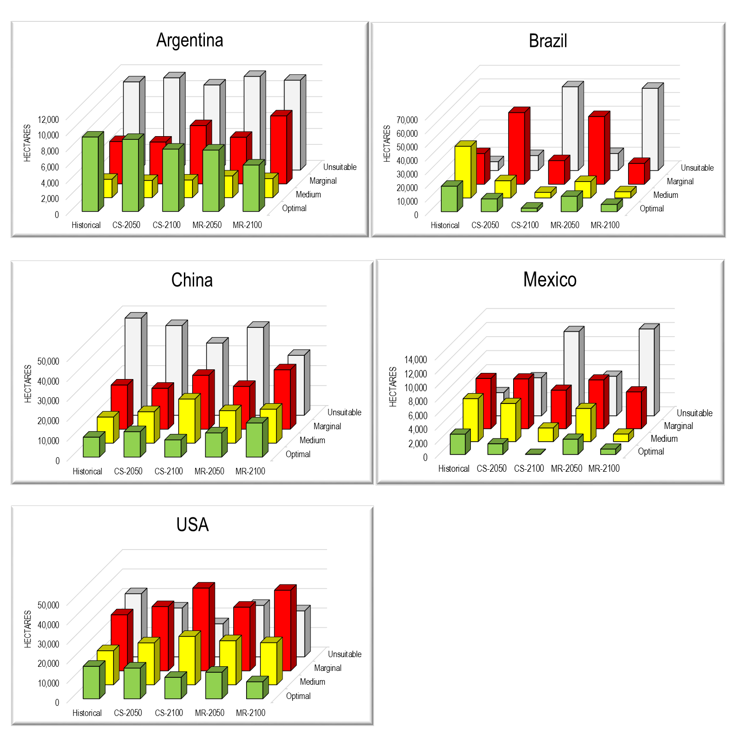


Figure S2. Projected unsuitable and suitable areas for the five major producers of maize under current and future climate conditions based on CS and MR models and the A2 scenario through CLIMEX


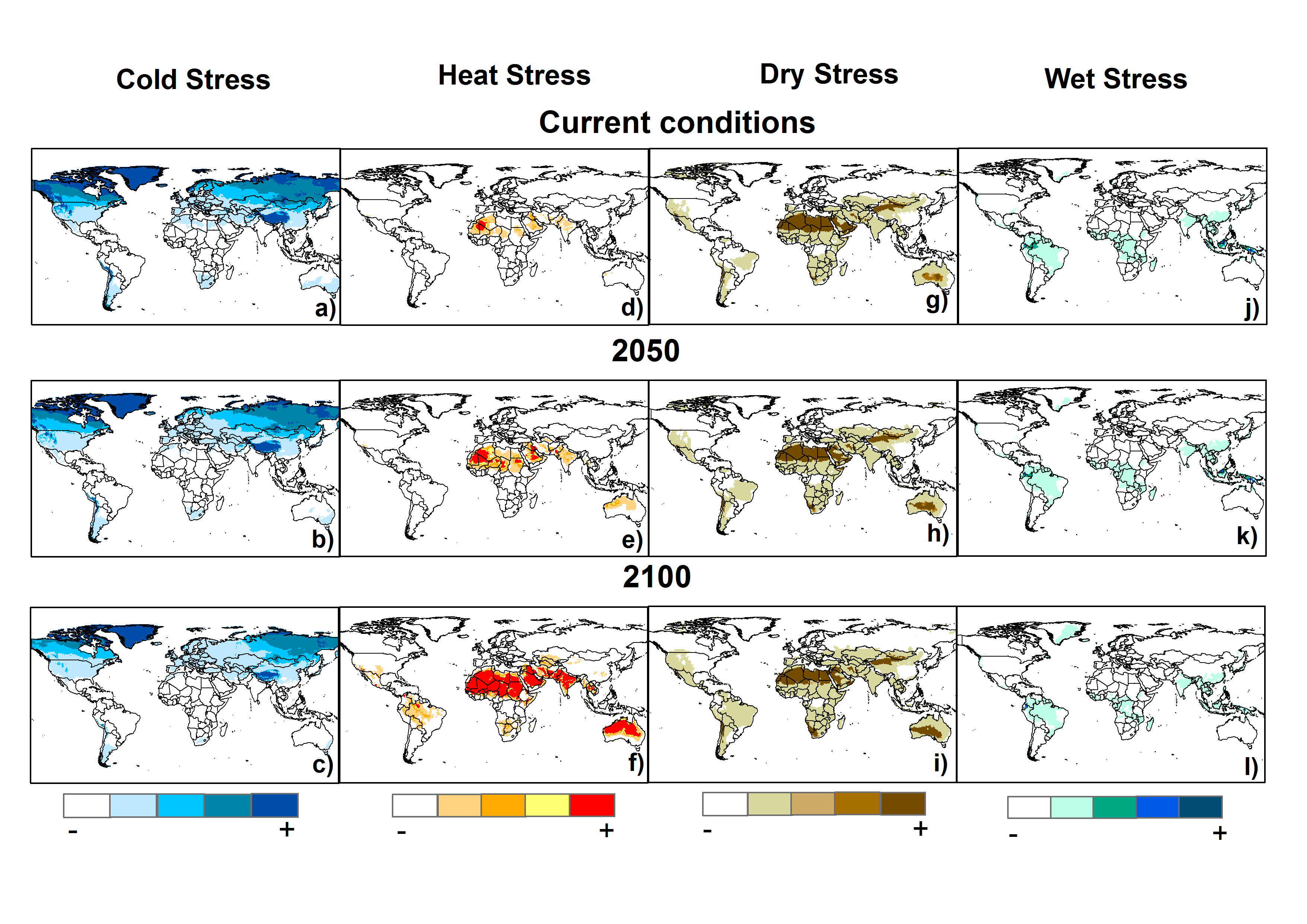


Fig S3. Projections of cold stress a) Current scenario, b) 2050, c) 2100; heat stress d) Current scenario, e) 2050, f) 2100; dry stress g) Current scenario, h) 2050, i) 2100 and wet stress j) Current scenario, k) 2050, l) 2100. ArcMap 10.2(http://desktop.arcgis.com/en/arcmap).

Table S1. CLIMEX sensitivity analysis values for maize (*Zea mays* L).

| **Parameter** | | **Parameter values** | | | **Change (%)** | | | | | | | | |
| --- | --- | --- | --- | --- | --- | --- | --- | --- | --- | --- | --- | --- | --- |
|  |  |  |  |  | **Range** | **EI** | **Growth index** | **Moisture index** | **Temp. index** | **Stress** | | | |
|  |  | **Low** | **Default** | **High** |  |  |  |  |  | **Cold** | **Heat** | **Dry** | **Wet** |
| Limiting low moisture | SM0 | 0 | 0.1 | 0.2 | 5.3 | 3.1 | 4.1 | 12.02 | 0 | 0 | 0 | 0 | 0 |
| Cold Stress Temperature Rate | THCS | -0.000084 | -0.00007 | -0.000056 | 3.5 | 1.1 | 0 | 0 | 0 | 10.28 | 0 | 0 | 0 |
| Dry Stress Threshold | SMDS | 0 | 0.1 | 0.2 | 2.3 | 2.2 | 0 | 0 | 0 | 0 | 0 | 49.57 | 0 |
| Cold Stress Temperature Threshold | TTCS | 6 | 7 | 8 | 2.2 | 0.9 | 0 | 0 | 0 | 8.72 | 0 | 0 | 0 |
| Limiting high moisture | SM3 | 1.2 | 1.3 | 1.4 | 1.2 | 2.5 | 2.7 | 8.76 | 0 | 0 | 0 | 0 | 0 |
| Lower optimal moisture | SM1 | 0.6 | 0.7 | 0.8 | 0.6 | 2.1 | 2.6 | 8.09 | 0 | 0 | 0 | 0 | 0 |
| Limiting high temperature | DV3 | 34 | 35 | 36 | 0.5 | 1.2 | 1.3 | 0 | 5 | 0 | 0 | 0 | 0 |
| Lower optimal temperature | DV1 | 17 | 18 | 19 | 0.4 | 1 | 1.5 | 0 | 3.7 | 0 | 0 | 0 | 0 |
| Limiting low temperature | DV0 | 9 | 10 | 11 | 0.3 | 0.6 | 0.9 | 0 | 1.7 | 0 | 0 | 0 | 0 |
| Wet Stress Threshold | SMWS | 1.2 | 1.3 | 1.4 | 0.3 | 0.5 | 0 | 0 | 0 | 0 | 0 | 0 | 13.42 |
| Upper optimal moisture | SM2 | 0.8 | 0.9 | 1 | 0.2 | 1.5 | 1.7 | 6.72 | 0 | 0 | 0 | 0 | 0 |
| Heat Stress Temperature Threshold | TTHS | 39 | 40 | 41 | 0.2 | 0.2 | 0 | 0 | 0 | 0 | 15.26 | 0 | 0 |
| Dry Stress Rate | HDS | -0.0108 | -0.009 | -0.0072 | 0.2 | 0.3 | 0 | 0 | 0 | 0 | 0 | 6.27 | 0 |
| Upper optimal temperature | DV2 | 29 | 30 | 31 | 0.1 | 1.6 | 1.8 | 0 | 7.5 | 0 | 0 | 0 | 0 |
| Heat Stress Temperature Rate | THHS | 0.008 | 0.01 | 0.012 | 0 | 0 | 0 | 0 | 0 | 0 | 3 | 0 | 0 |
| Wet Stress Rate | HWS | 0.0008 | 0.001 | 0.0012 | 0 | 0.2 | 0 | 0 | 0 | 0 | 0 | 0 | 2.26 |
